# Prognostic value of pretreatment inflammatory markers in localised prostate cancer before radical prostatectomy

**DOI:** 10.1007/s00345-023-04569-8

**Published:** 2023-09-25

**Authors:** Josias Bastian Grogg, Gianluca Rizzi, Jana Gadient, Marian Severin Wettstein, Andres Affentranger, Christian Daniel Fankhauser, Daniel Eberli, Cédric Poyet

**Affiliations:** Department of Urology, University Hospital of Zurich, University of Zurich, Frauenklinikstrasse 10, 8091 Zurich, Switzerland

**Keywords:** Urology, Oncology, Prostate cancer, Inflammatory markers, Prospective cohort

## Abstract

**Purpose:**

There is growing evidence of an association between inflammatory processes and cancer development and progression. In different solid tumor entities, a pronounced inflammatory response is associated with worse oncological outcome. In this study, we aim to evaluate the prognostic role of clinically established pretreatment inflammatory markers in patients with localised prostate cancer (PCa) before radical prostatectomy (RP).

**Methods:**

A total of 641 men met our inclusion criteria and were followed prospectively for a median of 2.85 years. Univariable logistic and Cox regression analysis were performed to analyse associations between preoperative inflammatory markers and tumor characteristics, and biochemical recurrence free survival (BRFS).

**Results:**

Median age at RP was 64 years. Gleason Score (GS) 7a (263, 41%) was the most prevalent histology, whereas high-risk PCa (≥ GS 8) was present in 156 (24%) patients. Lympho-nodal metastasis and positive surgical margin (PSM) were detected in 69 (11%) and 180 (28%) patients, respectively. No statistically relevant association could be shown between pretreatment inflammatory markers with worse pathological features like higher tumor stage or grade, nodal positive disease or PSM (for all *p* > 0.05). Additionally, pretreatment inflammatory markers were not associated with a shorter BRFS (*p* > 0.05). Known risk factors (tumor grade, tumor stage, nodal positivity and positive surgical margins) were all associated with a shorter BRFS (for all *p* < 0.0001).

**Conclusion:**

In this large prospective cohort, preoperative inflammatory markers were not associated with worse outcome.

**Supplementary Information:**

The online version contains supplementary material available at 10.1007/s00345-023-04569-8.

## Introduction

Prostate cancer (PCa) is the second most diagnosed cancer in men and accounts for approximately 7% of all cancer deaths in males. Worldwide, each year approximately 1.3 million men are diagnosed with PCa and it is the 5th leading cause of cancer death in men, nearly 360,000 men per year die because of it [3].

The association of inflammation and cancer is not new but the exact mechanisms in cancer development are not fully understood yet. In 1863 already, Virchow described cancer development through inflammatory processes [2]. Approximately 15% of all oncologic diseases are linked to chronic inflammation and/or infection [9]. The association of different inflammatory markers and carcinogenesis and oncological outcome has been investigated thoroughly. One of the most studied inflammatory marker is the neutrophil-to-lymphocyte ratio (NLR). The association of a high NLR with worse oncological outcome in different tumor types, notably colorectal-, gastric-, lung, liver- and renal cancer, has been shown in systematic reviews and meta-analyses [5, 15]. Other inflammatory markers with significant associations to worse oncological outcome in different solid tumors include a high platelet-to-lymphocyte ratio (PLR) [14], and elevated C-reactive protein (CRP) [7]. However, true cutoff-values for NLR and PLR are lacking, which makes the comparison of study results challenging.

Chronic inflammatory disease and elevated systemic inflammatory markers have also been linked to PCa development [4]. For example, significantly higher PLR values were found in prostate cancer patients when compared with benign prostatic hyperplasia (BPH) patients or in the healthy control group [11]. Furthermore, there are studies showing evidence that high NLR is associated with worse outcome in PCa patients [1, 10, 18].

The aim of the present study was to evaluate the prognostic value of preoperative inflammatory markers, particularly neutrophils, NLR, PLR and CRP, in patients who underwent radical prostatectomy (RP) for localised PCa. We hypothesised that an elevated preoperative inflammatory state would predict a worse oncological outcome in terms of a shorter biochemical recurrence-free survival (BRFS).

## Material and methods

### Study design

Men with clinically localised PCa undergoing radical prostatectomy (RP) were prospectively enrolled in this single-center cohort study (*Pro*state *C*ancer *O*utcomes *C*ohort Study: ProCOC [16]).

The study was approved by the Ethics Committee of Canton Zurich (protocol name: ProCOC: The Prostate Cancer Outcomes Cohort Study, protocol number: Ref. Nr. StV KEK-ZH-Nr. 06/08). All men gave written informed consent. The ProCOC study is mainly designed to analyse clinicopathological and prognostic biomarkers, which would ultimately predict oncological outcome in men with localised PCa. For this study, lack of full blood count (> 30 days prior to surgery) was the main exclusion criteria. Furthermore, patients with secondary systemic inflammatory diseases, such as myeloproliferative disorders or secondary malignancies were also excluded.

Patients were normally followed on a regular basis for every three months during the first year, which refers to the first year after RP. Afterwards, follow-up took place at least annually or on an individual basis depending on the disease course. A prostate specific antigen (PSA) value ≥ 0.1 ng/ml during follow-up was defined as biochemical recurrence (BCR). Men were censored if lost to follow-up or event-free at their most recent clinic visit. Patients with a postoperative PSA persistence or without distinct follow-up data for the endpoint BCR were excluded from the analysis of BCR.

All surgical specimens were processed according to standard histopathological procedures and as previously described [17]. Tumor characteristics were obtained from pathology reports according to the World Health Organization/International Society of Urologic Pathologists (WHO/ISUP 2016) classification.

Based on our previous literature review and the laboratory parameters regularly measured at our institution, we defined the following preoperative inflammatory parameters as relevant for our analysis: neutrophil count, platelet count, lymphocytes, NLR, PLR and CRP.

Preoperative blood samples were processed by the hospital’s Institute of Hematology, CRP and PSA values were measured by the hospital’s Institute for Clinical Chemistry. Differential blood count was determined automatically and NLR and PLR were calculated as follows: $\mathrm{Neutrophil \, count }(\mathrm{G}/\mathrm{L})/\mathrm{Lymphocytes }(\mathrm{G}/\mathrm{L})$ and $\mathrm{Platelet \, count } (\mathrm{G}/\mathrm{L})/\mathrm{Lymphocytes }(\mathrm{G}/\mathrm{L})$, respectively.

Predefined laboratory testing, such as complete blood count, CRP- and PSA levels, serum lipid level and creatinine level, was performed mostly the day before surgery.

### Statistical analysis

Univariable logistic regression analysis was performed to investigate a possible association between preoperative inflammatory markers (neutrophil count, NLR, PLR and CRP) and adverse clinicopathological high-risk features (extraprostatic disease (≥ pT3), high-risk PCa (≥ Gleason Score 8), positive nodal disease (pN1) and positive surgical margins (PSM). All preoperative inflammatory markers were analysed as continuous and dichotomised variables (binary variables) with a cut-point at the median of all preoperative inflammatory markers. Kaplan–Meier analysis and univariable Cox proportional hazards regression analysis were used to evaluate the association between inflammatory markers and time to BCR or secondary therapy. Differences were considered statistically significant if *p* < 0.05 (two-sided). A shorter biochemical recurrence free survival (BRCS) is defined by a statistical significance of *p* < 0.05 in Cox proportional hazard regression analysis or Kaplan–Meier analysis.

Statistical analysis was performed using R 4.0.3 studio software (R Foundation for Statistical Computing, Vienna, Austria [6]).

## Results

### Patient characteristics

A total of 788 men receiving RP have been included in the ProCOC study between November 2008 and December 2019 and were screened for the study (Supplementary Fig. 1). A total of 134 (17%) patients were excluded due to incomplete or unavailable preoperative differential blood count. Twelve (1.5%) more men were excluded due to secondary systemic reasons for potential blood count modulation (chronic systemic inflammatory diseases: *n* = 4, inflammatory bowel diseases: *n*  = 2, immunosuppression: *n* = 2, myeloproliferative diseases: *n* = 3, other malignancies: *n*  = 1). Additionally, one (< 1%) patient had to be excluded as final histopathology of the prostate revealed no evidence of cancer.

The final cohort consisted of 641 men with a median age of 64 years. Median preoperative PSA level was 7.72 ng/mL. Histopathological analysis revealed extraprostatic extension in 217 (34%) cases and high risk PCa (≥ GS 8) was found in 156 (24%) men. Lympho-nodal metastasis and PSM were detected in 69 (11%) and 180 (28%) patients, respectively. GS 6 tumors were found in 34 (5%), GS7 in 451 (70%) and GS ≥ 8 in 156 (24%) patients, respectively. Median neutrophil count was 4.16 G/l, median NLR and PLR at 2.85 and 161, respectively (Supplementary Table 1). A more detailed data analysis on the distribution pattern of preoperative inflammatory markers for the entire cohort is shown in Supplementary Fig. 2.

### Association of inflammatory markers with clinicopathological features

Possible associations of preoperative inflammatory markers with clinicopathological high-risk features (tumor stage, tumor grade, nodal positive disease, PSM) was analysed in univariable logistic regression models (Table 1). All tested preoperative inflammatory markers did not show an association with extraprostatic disease (≥ pT3), high-risk prostate cancer (≥ GS8), positive nodal disease (pN1) and PSM, in both continuous and dichotomised analysis (all *p* > 0.05).

**Table 1:** Univariable logistic regression analysis to assess an association between pretreatment inflammatory markers and clinicopathological high-risk features

Inflammatory markers	Outcome (logistic regression analysis)							
	≥ pT3		≥ Gleason 8		pN1		PSM	
	OR (95% CI)	*p *value	OR (95% CI)	*p *value	OR (95% CI)	*p *value	OR (95% CI)	*p *value
Neutrophil count (continuous)	0.99 (0.88-1.11)	0.82	1.11 (0.99-1.26)	0.08	0.87 (0.71-1.05)	0.16	1.02 (0.90-1.15)	0.76
Neutrophil count (dichotomized)	0.79 (0.57-1.09)	0.15	1.07 (0.74-1.53)	0.73	0.65 (0.39-1.08)	0.10	0.88 (0.62-1.24)	0.47
Neutrophil-to-lymphocyte ratio (continuous)	0.99 (0.88-1.12)	0.90	1.09 (0.96-1.24)	0.19	0.90 (0.73-1.09)	0.31	1.01 (0.89-1.14)	0.90
Neutrophil-to-lymphocyte ratio (dichotomized)	0.95 (0.69-1.32)	0.78	1.18 (0.82-1.70)	0.37	0.70 (0.42-1.15)	0.16	1.00 (0.71-1.41)	0.98
Platelet-to-lymphocyte ratio (continuous)	1.00 (1.00-1.00)	0.88	1.00 (1.00-1.00)	0.65	1.00 (1.00-1.00)	0.97	1.00 (1.00-1.00)	0.96
Platelet-to-lymphocyte ratio (dichotomized)	1.04 (0.75-1.44)	0.82	1.14 (0.79-1.64)	0.48	1.10 (0.67-1.82)	0.71	1.09 (0.77-1.54)	0.62
CRP (continuous)	1.02 (0.99-1.05)	0.25	1.03 (0.99-1.06)	0.12	1.03 (0.99-1.07)	0.12	1.00 (0.96-1.03)	0.92
CRP (dichotomized)	1.10 (0.79-1.53)	0.56	1.20 (0.84-1.73)	0.32	1.07 (0.65-1.77)	0.80	1.16 (0.65-1.77)	0.41

≥ pT3: extraprostatic disease; Gleason ≥ high-risk disease

*pN1* nodal positive disease, *PSM* positive surgical margins, *OR* odds ratio, *Cl* confidence interval

### Prediction of biochemical recurrence free survival after radical prostatectomy

From the 641 patients included in the final cohort, a total of 536 patients (84%) were included for survival analysis of BCR. A total of 105 patients (16%) were additionally excluded from this analysis for the following criteria: 92 (14%) men did not reach a PSA nadir below < 0.03, in six patients (1%) no postoperative PSA was available and another seven (1%) patients received direct adjuvant therapy without evidence of biochemical recurrence (Supplementary Fig. 1). Median follow-up time in these 536 patients was 2.85 years (interquartile range 1.48–4.67 years).

To evaluate the prognostic significance of pretreatment inflammatory markers and BRFS, an univariable Cox regression analysis was performed. Patient age at diagnosis, preoperative neutrophils, NLR, PLR and CRP as continuous or dichotomised variables showed no statistical association with BCR. On the other hand, extraprostatic disease, high GS, nodal positive disease and PSM, as well as preoperative PSA level were strongly associated with BCR (*p* < 0.0001).These results are summarised in Table 2.

**Table 2:** Univariable Cox regression to analyse different prediction markers for biochemical recurrence

Variables	*n* (%)^a^	Events (%)^b^	Biochemical recurrence	
			HR (95%-CI)	*p *value
Age (continuous)			0.99 (0.96-1.02)	0.49
PSA (continuous)			1.03 (1.02-1.05)	**<0.0001**
Extraprostatic disease (≥ pT3 vs. lower)	139 (25.9)	37 (26.6)	3.45 (2.19-5.42)	**<0.0001**
High-risk disease (≥ Gleason 8 vs. lower)	100 (18.7)	35 (35.0)	4.61 (2.93-7.26)	**<0.0001**
Nodal positive disease	24 (4.5)	12 (50.0)	7.95 (4.20-15.03)	**<0.0001**
Positive surgical margins (yes vs. no)	116 (21.6)	38 (32.8)	3.64 (2.32-5.71)	**<0.0001**
Neutrophil count (continuous)			0.92 (0.77-1.10)	0.37
Neutrophil count (dichtomotomized)	275 (51.3)	41 (14.9)	1.08 (0.69-1.69)	0.75
Neutrophil-to-lymphocyte ratio (continuous)			0.87 (0.72-1.05)	0.15
Neutrophil-to-lymphocyte ratio (dichotomized)	270 (50.4)	36 (13.3)	0.82 (0.52-1.28)	0.38
Platelet-to-lymphocyte ratio (continuous)			1 .00 (0.99-1.00)	0.16
Platelet-to-lymphocyte ratio (dichotomized)	262 (48.9)	36 (13.7)	0.89 (0.56-1.39)	0.60
CRP (continuous)			0.88 (0.77-1.01)	0.06
CRP (dichotomized)	266 (49.6)	38 (14.3)	0 .99 (0 .63-1.55)	0.96

Bold faces representing significant values (*p* < 0.05)

*PSA* prostate specific antigen, *HR* hazard ratio, *Cl* confidence interval, *CRP* C-reactive protein

^a^Number and percent of patients above the cut-point

^b^Number and percent of patients with biochemical recurrence

**Fig. 1:**
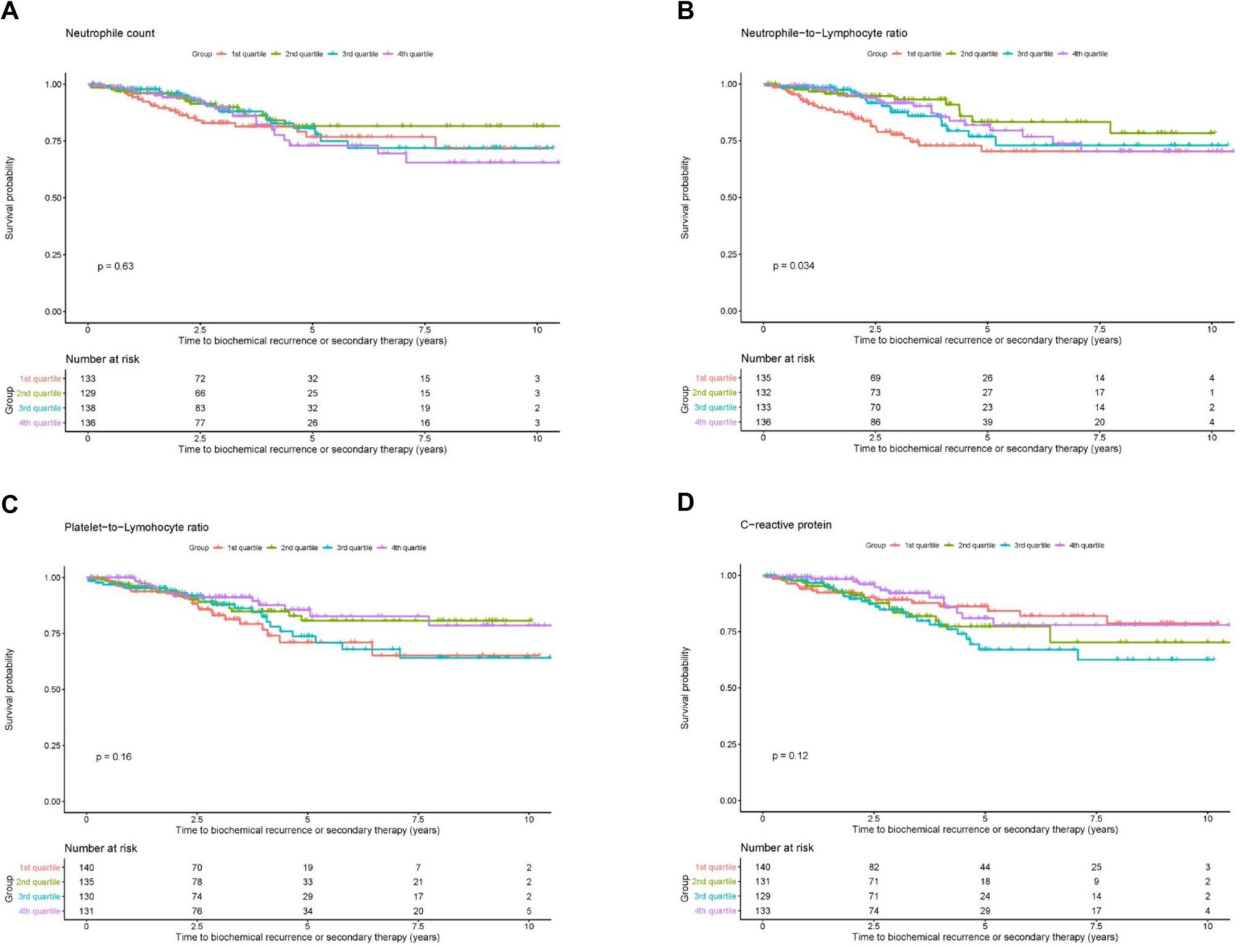
Kaplan–Meier curve and corresponding log rank test show relation of biochemical recurrence with **A** neutrophil count, **B** neutrophil-to-lymphocyte ratio (NLR), **C** platelet-to-lymphocyte ratio (RLR), **D** C-reactive protein (CRP)

Additionally, Kaplan–Meier estimates and corresponding log-rank test were performed for further visualisation of the correlation between BCR and neutrophils (Fig. 1A), NLR (B), PLR (C) and CRP (D). All inflammatory markers were tested as continuous and dichotomized variables at different cut-off points (quartiles: 25%, 50% and 75%). All tested preoperative inflammatory markers did not show a statistically relevant association with BRFS (for all *p* > 0.05), whereas known pathological adverse factors (extraprostatic disease, tumor grade, nodal positive disease and surgical margin) all showed a significant shorter BRFS (*p* < 0.05) (Supplementary Fig. 3).

## Discussion

In this study, we assessed the value of preoperative inflammatory markers in association to known clinicopathological features and outcome in 641 patients with localised PCa, who underwent RP. In our prospective analysis, preoperative inflammatory markers, including neutrophil count, NLR, PLR and CRP, did not show any association with high-risk clinicopathological features and inflammatory markers were not able to predict a shorter BRFS. Thus, pretreatment inflammatory markers seem not clinically relevant in men with localised PCa.

On the other hand, all known histopathological adverse factors including extraprostatic disease, high GS, nodal positive disease and PSM, as well as preoperative PSA level were strongly associated with BCR. We consider this to be an indicator of quality of our methodology and data analysis. Furthermore, these results underline the reproducibility of our cohort and reiterate the prognostic value of these clinic-pathological risk factors.

Nevertheless, our study results conflict with some of the literature. A retrospective study by Jang et al. reviewed medical reports of 2′301 men with localised PCa undergoing RP. They found a significant association of pretreatment NLR ≥ 1.76 with decreased OS (*p* = 0.003) and decreased CSS (*p* = 0.005) but not for decreased BRFS (*p* = 0.223). Similar to us, they used a median cut-point for NLR. On uni- and multivariable regression analysis, NLR was a predictor of cancer-specific survival (CSS) (hazard ratio (HR) 2.012, 95% confidence interval (CI) 1.222–3.310, *p* = 0.006) and overall survival (OS) (HR 1.650, 95% CI 1.127–2.416, *p* = 0.010) [8].

Conversely, more recent publications showed results that are in line with our findings. A meta-analysis from Tang et al. from 2016 included 18 studies with a total of 9418 men with PCa (localised) and locally advanced/metastatic, treated with chemotherapy, RP, or radiotherapy [13]. Pretreatment NLR was not associated with OS in the subgroup of patients with localised PCa (HR 1.439, 95% CI 0.753–2.75) but showed significant association for OS (HR 1.628, 95% CI 1.410–1.879) and BRFS (HR 1.357, 95% CI 1.126–1.636) in all patients with PCa taken together [14].

Different studies have evaluated pretreatment inflammatory markers in *advanced* PCa and mCRPC. A recent meta-analysis performed by Peng et al. investigated a possible association of established pretreatment inflammatory markers with cancer outcome and included 32 studies in total. They included localised PCa patients, metastatic castration resistant prostate cancer (mCRPC) and CRPC patients with different treatment modalities and stated that high pretreatment inflammatory markers predict inferior OS outcomes. A shorter BRFS was shown for higher level of NLR but not for neutrophil count. There was no association of NLR and neutrophil count with shorter CSS after treatment, however. For PLR, no association was seen with shorter CSS after treatment but shorter OS (HR = 1.72; 95% CI 1.36–2.18, *p* < 0.001). There was a significant heterogeneity among the chosen studies [12]. In summary, it seems that advanced or metastatic PCa triggers a more systemic inflammation response than localised disease. However, in this study we focused our analysis exclusively on patients with localised PCa, therefore no conclusions can be drawn for more advanced disease.

In summary, the available evidence for the prognostic value of pretreatment inflammatory markers in localised PCa is scarce and shows conflicting results. Specifically, most of the reported studies lack a clear statistical concept or are based on a rather small sample size. Additionally, different arbitrary cut-off points for NLR, derived-NLR and PLR were used in the published literature, which limit the comparability. In our study, we did not search for the optimal cut-point but rather looked at an association between inflammatory markers as continuous variables and cut-points at the median and quartiles of the entire cohort.

The present study has several limitations. Firstly, the median follow-up time in our cohort was relatively short with a median follow-up time of 2.85 years. Secondly, our primary endpoint was BCR free survival, which is not always a direct surrogate marker for CSS and OS. No statement about implications on OS or CSS can therefore be made. Furthermore, differential blood count was only determined once before RP, which does not allow assessing the dynamic of this inflammatory marker during follow-up after RP.

To the best of our knowledge, this study represents the largest and most comprehensive analysis in a well-characterised cohort evaluating all commonly used inflammatory markers in the setting of localised PCa in a prospective manner.

Our data suggest no additional value of pretreatment inflammatory markers in patients with localised PCa. We therefore conclude that localised PCa does not trigger a measurable systemic inflammation response.

### Supplementary Information

Below is the link to the electronic supplementary material.

Supplementary file 1 (ZIP 521 KB)

## Data Availability

The datasets generated and analysed during the current study are available from the corresponding author on reasonable request.

## Abbreviations

BCR: Biochemical recurrence
BPH: Benign prostatic hyperplasia
BRFS: Biochemical recurrence free survival
CI: Confidence interval
CRP: C-reactive protein
CSS: Cancer specific survival
GS: Gleason Score
HR: Hazard ratio
mCRPC: Metastatic castration resistant prostate cancer
NLR: Neutrophil-to-lymphocyte ratio
OS: Overall survival
PCa: Prostate cancer
PLR: Platelet-to-lymphocyte ratio
PSA: Prostate specific antigen
PSM: Positive surgical margins
RP: Radical prostatectomy
WHO/ISUP: World Health Organization/International Society of Urologic Pathologists
