# Supplementary material for: Prognostic value of pretreatment inflammatory markers in localised prostate cancer before radical prostatectomy
Source: World J Urol. 2023 Sep 25;41(10):2693–8. doi: 10.1007/s00345-023-04569-8 (PMC10581955; doi:10.1007/s00345-023-04569-8)
Supplement: Supplementary file 1 — (ZIP 521 KB) [file 345_2023_4569_MOESM1_ESM.zip › 07 Suppl. Table 1.docx]

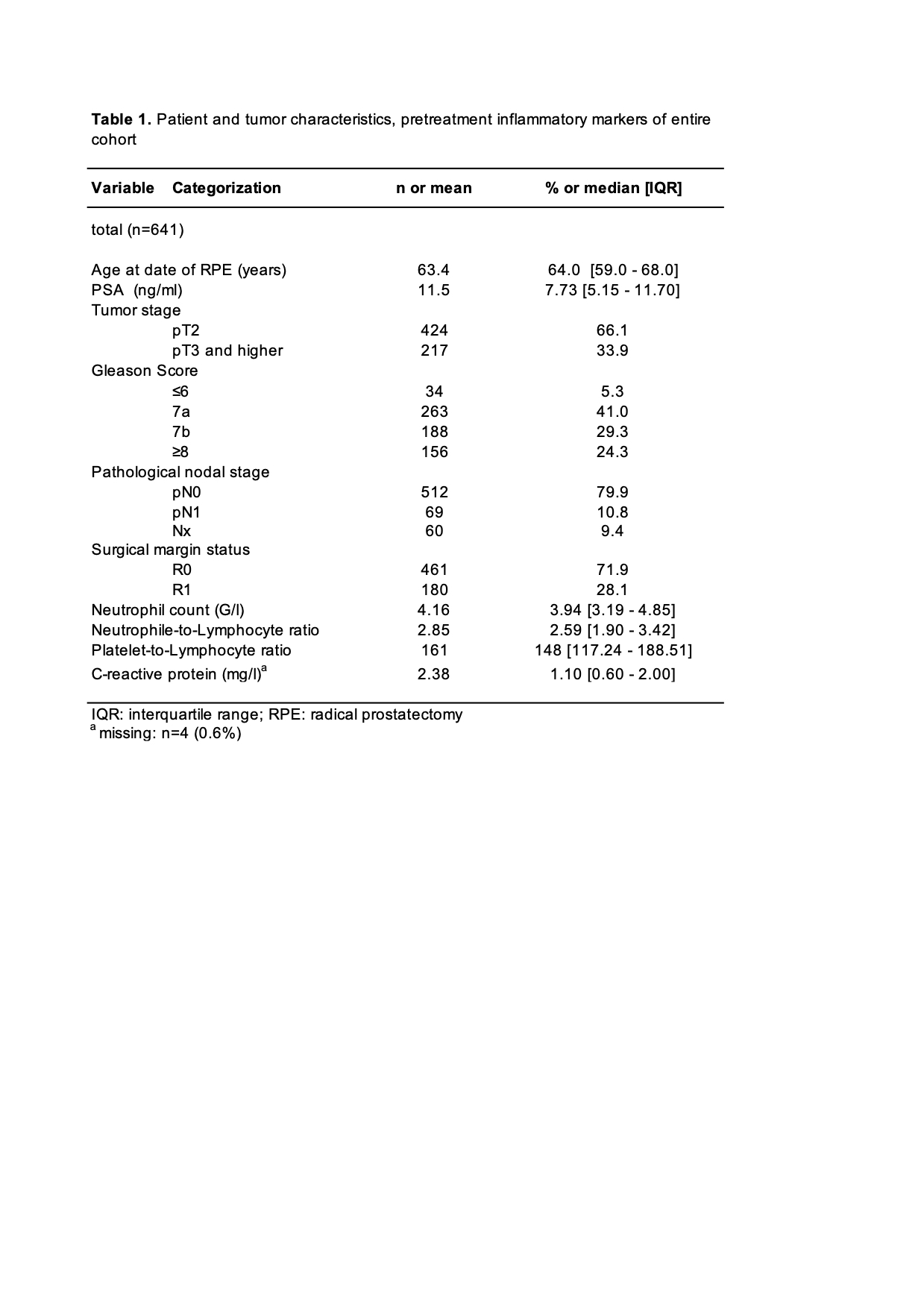


**Supplementary Table 1:** Patient and tumor characteristics, pretreatment inflammatory markers of entire cohort
