# Supplementary material for: Prognostic value of pretreatment inflammatory markers in localised prostate cancer before radical prostatectomy
Source: World J Urol. 2023 Sep 25;41(10):2693–8. doi: 10.1007/s00345-023-04569-8 (PMC10581955; doi:10.1007/s00345-023-04569-8)
Supplement: Supplementary file 1 — (ZIP 521 KB) [file 345_2023_4569_MOESM1_ESM.zip › 06 Suppl. Figure 3.docx]

| **A**  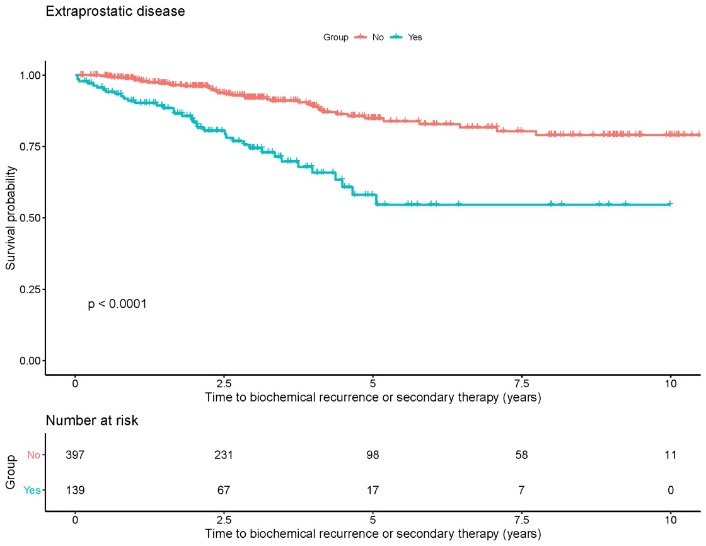  **C**  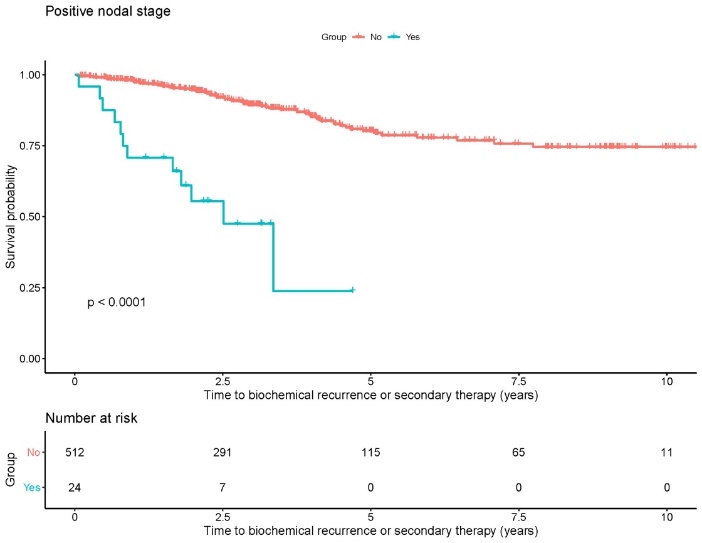 | **B**  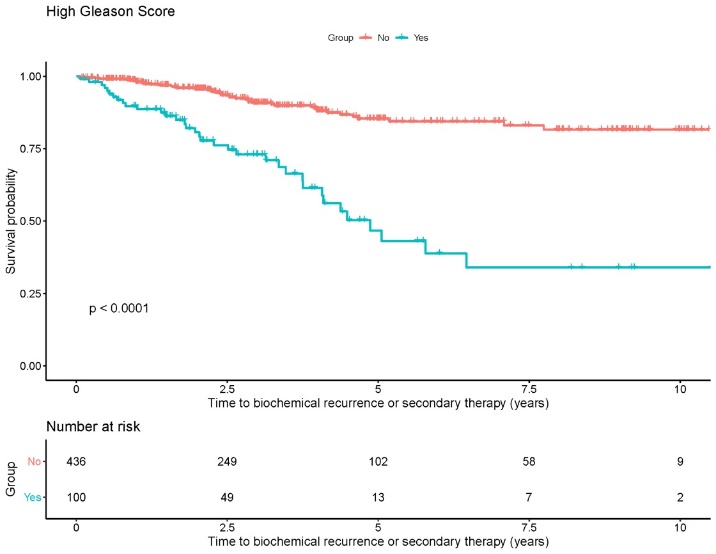  **D**  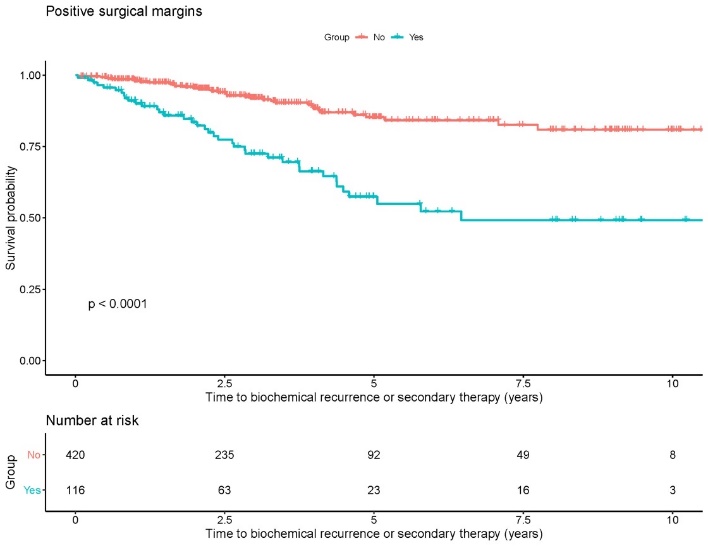 |
| --- | --- |

**Supplementary Figure 3** : Kaplan-Meier curve and corresponding log rank test show prognostic association of biochemical recurrence and (A) extraprostatic disease (tumorstage ≤ T2c vs. ≥ T3a ), (B) tumor grade (≤ Gleason Score 7b), (C) nodal positive disease (pN0 vs pN1) and (D) surgical margin (R0 vs R1)
