# Supplementary figures and images for: Prognostic value of pretreatment inflammatory markers in localised prostate cancer before radical prostatectomy
Source: World J Urol. 2023 Sep 25;41(10):2693–8. doi: 10.1007/s00345-023-04569-8 (PMC10581955; doi:10.1007/s00345-023-04569-8)

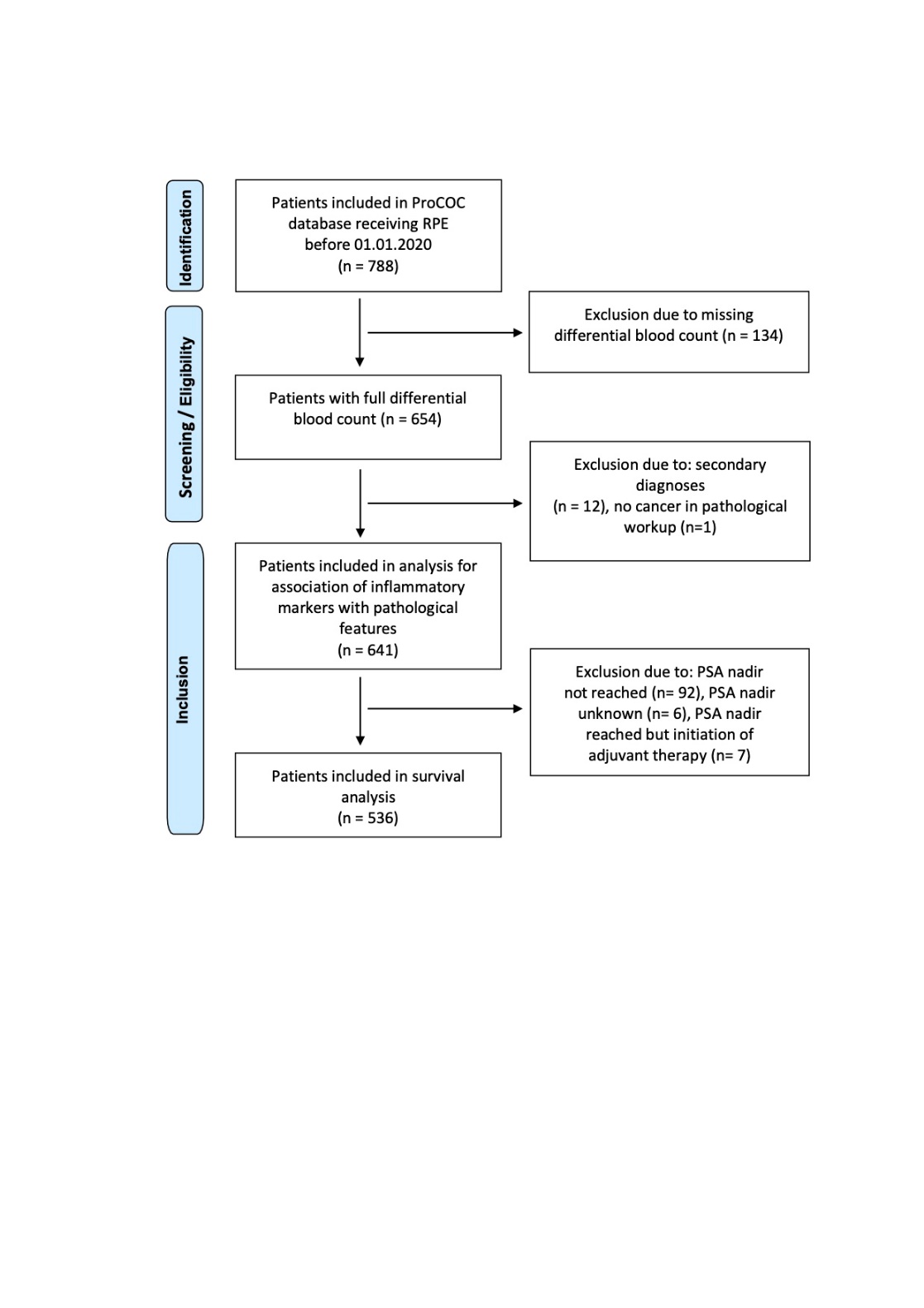


**Supplementary Figure 1:** Flowchart of patient identification, screening and inclusion

Supplement: Supplementary file 1 — (ZIP 521 KB) [file 345_2023_4569_MOESM1_ESM.zip › 04 Suppl. Figure 1.docx]
